# Video-assisted radiofrequency ablation for pleural disseminated non-small cell lung cancer

**DOI:** 10.1186/1471-2482-13-19

**Published:** 2013-06-13

**Authors:** Yaxing Shen, Ming Zhong, Wei Jiang, Hong Fan, Hao Wang, Qun Wang

**Affiliations:** 1 Division of Thoracic Surgery, Zhongshan Hospital, Fudan University, Shanghai, China; 2 Department of Anesthesiology, Zhongshan Hospital, Fudan University, Shanghai, China

**Keywords:** Ablative therapy, Lung cancer surgery, Thoracoscopy/VATS

## Abstract

**Background:**

Clinically, some patients would have false-negative results in the diagnosis of non-small cell lung cancer (NSCLC) with pleural dissemination, losing their chances of prolonged survival from surgery. Hence, this study aimed to clarify the benefit of radiofrequency ablation (RFA) for NSCLC with malignant pleural dissemination that is detected during thoracoscopic lobectomy.

**Methods:**

From July 2006, we started the application of RFA in combination with talc pleurodesis (R-TP) for pleural disseminated NSCLCs diagnosed by thoracoscopy. Patients who underwent TP alone (from December 30, 2005 to June 30, 2006) were retrospectively evaluated in compared with R-TP (from July 1, 2006 to June 30, 2008). Clinical features were collected and compared to identify the difference in clinical outcomes between R-TP and TP alone. After discharge (three months after surgery), tumor response to treatment was assessed, and follow-up results were recorded to determine the perioperative and mid-time survival difference between the two groups.

**Results:**

In our study, the two groups were comparable in age, sex, performance status (PS) score, tumor location, and histological diagnosis. The incidence rate of intraoperative pleural dissemination was 5.98%, as diagnosed by video-assisted thoracoscopy. All the surgeries were completed without conversion to open thoracotomy. Except for the longer operation duration in the R-TP group (*p* < 0.001), there was no significant difference between the two groups in terms of surgical features. Postoperatively, no mortality occurred in either group during hospital stay; however, two patients from the R-TP group developed complications (9.52%). The complete and partial remission rates in the R-TP group were 80% and 10%, respectively, and the stabilization rate was 10%. After the three-year follow-up, the overall survival (OS) rates of the R-TP and TP groups were 14.29% and 0%, respectively. The median survival and median tumor progression-free survival (PFS) periods were longer in the R-TP group than in the TP group (OS: 19 months versus 12.5 months, *p* = 0.045; PFS: 9.5 months versus 5.5 months, *p* = 0.028).

**Conclusions:**

The introduction of RFA to TP offered survival benefits to pleural disseminated NSCLC patients, making it a potential alternative palliative treatment for local tumor. However, multicenter randomized controlled trials are required to confirm these findings.

## Background

Curative resection is the standard treatment for primary (stage I–IIIa) non-small cell lung cancer (NSCLC) [1]. However, some cases diagnosed as “early-stage” cancer before surgery are actually found to be pleural disseminated NSCLC during the operation. For patients with such cases, surgical resection is not the treatment of choice [2]. However, the prognosis would remain poor even if the patient undergoes chemotherapy or radiotherapy instead, with a survival rate close to stage IIIa–IIIb cancer, as reported by Sawabata and colleagues [3]. Traditionally, talc pleurodesis (TP) is performed for the control of pleural effusions associated with pleural disseminated NSCLCs [4]. Although TP has been shown to be safe and effective, the prognoses of patients who undergo TP remain unchanged. Hence, new treatment strategies are required to improve the survival of patients with advanced-stage NSCLC.

Since first reported by Dupuy and colleagues, radiofrequency ablation (RFA) has been considered an alternative treatment of lung cancer [5] and shown to obtain remarkable outcomes especially in patients with early-stage cancer who could not tolerate standard surgery. In the current literature, the reported complete remission (CR) rate, as well as the median survival time from NSCLC patients underwent RFA were promising [6-9]. However, as for advanced lung cancers, whether RFA could obtain the same remarkable outcomes as it did for early-stage lesions remains unclear.

In this study, we hypothesized that RFA is beneficial to patients with pleural disseminated NSCLC. Therefore, we compared alternative (RFA combined with TP [R-TP]) and conservative therapies (TP alone) to determine whether R-TP would be technically safe and oncologically beneficial in the treatment of advance NSCLCs.

## Methods

At our institution, lung cancer is clinically staged by thoracic computed tomography (CT), abdominal ultrasound, brain MRI, and isotopic bone scanning. According to the UICC system, In this study, the inclusion criteria for VATS lobectomy are:

a. patients with clinical staged T1–T3N0M0 lung cancer;

b. patients without cancer history;

c. patients with no previous history of chest surgery;

d. patients with normal lung and heart function;

e. patients with an ASA score of I-III.

In case contraindications are found during thoracoscopic exploration, a conversion from radical to palliative surgery would be necessary. Conventionally, when pleural dissemination is verified during VATS, TP would then be performed. Since July 1, 2006, TP was substituted by R-TP at our institution. The exclusion criteria for R-TP included central tumors (within 3 cm of the hilum) and refusal to undergo R-TP. In this retrospective study, the clinical features and survival results of the R-TP group (from July 1, 2006, to June 30, 2008) were collected and compared with those of the TP group (from December 30, 2005 to June 30, 2006) to identify the difference between the two groups of patients.

Written informed consent was obtained from all the patients before undergoing R-TP. The ethics committee of Zhongshan hospital of Fudan university approved this research and granted a waiver for individual patient consent for those patients who were retrospectively evaluated.

### Operation

#### Radiofrequency ablation

All the patients underwent a combination of epidural and general anesthesia. During the operation, a double-lumen endotracheal tube was used to selectively deflate a single lung, with a continuous positive airway pressure of ten mmHg. The pleural tumor dissemination was verified by frozen sectioning. RFA was performed by using the RITA system(RITA Medical Systems Inc, USA). The deployment of the electrosurgical needle was staged according to the size of the tumor. Under thoracoscopic visual guidance, the probes were inserted 0.5 cm into the primary tumor, and multiple electrodes were deployed to two centimeters within the tumor (sequentially to three, four, and five centimeters). Thermal ablation was extended for 15 minutes after the tumor temperature reached 90°C. In the case of a large tumor (diameter longer than three centimeters), multiple repositioning and reablation at different sites within the tumor were required to ensure ablation of the entire lesion. Then six–eight grams of sterilized talc powder was sprayed into the pleural cavity. With a chest tube for drainage, the surgery concluded with closure of the incisions in layers. In TP, when the pathologists concurred on the diagnosis of pleural dissemination, the procedure was to spray six–eight grams of talcum powder into the pleural cavity and close the incision, using a chest tube for drainage.

#### Follow-up

Postoperatively, all the patients were followed up until December 2010 by CT scans, abdominal type-B ultrasonic examination, and brain MRI inspection every three months. An isotopic bone scan was obtained every six months. Tumor response to treatment was assessed three months after surgery, according to the modified Response Evaluation Criteria in Solid Tumors.

#### Data collection and statistical analysis

The clinical data including patient demographics, tumor characteristics, surgical features, and clinical outcomes of both groups were collected from the clinical database of the Thoracic Division of the Zhongshan Hospital, Fudan University.

The information described earlier was recorded in Excel for further processing. Statistical analysis was performed with the SPSS software (version 17.0), using the *t* test and chi-square test. Continuous variables were compared using the Mann–Whitney test. A two-sided *p* < 0.05 was considered statistically significant.

## Results

### Clinical features

From December 2005 to June 2008, 752 NSCLC patients were eligible for VATS at our institution, 45 (5.98%) of whom demonstrated pleural dissemination during the operation. In our study, we performed R-TP for 21 pleural disseminated NSCLC patients until June 2008 and 24 patients who underwent TP were enrolled as historical controls. The incidence of unexpected pleural dissemination was close between R-TP and TP (21/339 versus 24/413, *p* = 0.832). In terms of patient demographics, the two groups had comparable results in age, sex, PS score, tumor volume, location, and histological diagnosis (listed in Table 1).

**Table 1:** Patient demographics

	**R-TP**	**TP**	***p *Value**
No.	21	24	
Age (y)	61.5 ± 3.2	59.7 ± 4.4	0.787
Sex			0.632
Male	12	12	
Female	9	12	
PS			0.055
0	13	8	
1	8	16	
Histological diagnosis			0.062
AD	19	22	
SQ	2	0	
AS	0	2	0.778
Tumor diameter (cm)	3.13 ± 1.01	2.80 ± 0.86	
Postoperative treatment			0.846
Chemotherapy	10	18	
Targeted therapy	2	3	
Supportive therapy	9	3	

*AD* adenocacinoma, *SQ* squamous carcinoma, *AS* adenosquamous carcinoma.

All the surgeries were completed without conversion to open thoracotomy. However, two postoperative complications were recorded for R-TP: one patient developed pneumonia, which was later cured by conservative therapy, and the other patient had a case of pneumothorax but recovered after a three-day chest drainage. We did not find similar cases for TP. The median hospital stay was eight days (range, 7–14 days) for the R-TP group and six days for the TP group (range, 4–16 days). As for the surgical characteristics, the operation duration in the R-TP group was longer than that in TP group (Tables 1 and 2). After surgery, five patients (two patients in TP and three patients in R-TP) were given targeted therapy orally (Gefitinib or Erlotinib) due to gene analysis for EGFR mutation, 28 patients (16 patients in TP and 12 patients in R-TP) received chemotherapy (platin-based chemotherapy, eg: Docetaxel plus Cisplatin) for four-six cycles, and 12 patients (seven patients in TP and five patients in R-TP) received supportive therapy including pain control, pleural drainage or nutrition support.

**Table 2:** Perioperative characteristics

**Perioperative characteristics**	**R-TP**	**TP**	***p *Value**
Surgery duration (min)	110 (60–175)	50 (45–85)	<0.001
Blood loss (mL)	80 (40–120)	60 (20–90)	0.227
Blood transfusion	0	0	
Duration of drainage (days)	2.5 (2–3)	3(2–5)	0.143
Total drainage amount (mL)	105 (60–360)	80 (0–390)	0.069
Length of stay (days)	8 (7–14)	6 (4–16)	0.884
Mortality	0	0	
Morbidity	2	0	

The surgery duration includes intraoperative pathological consultation.

### Survival

In the R-TP group, 20 patients survived for three months after the surgery and one patient died owing to systemic infection after chemotherapy. The CT scans showed that 16 patients (80%) had complete remission, two (10%) had partial remission, and two (10%) remained stable. The total effective rate (CR + PR) was 90%.

After the three-year follow-up, tumor progression occurred in 19 patients in the R-TP group, including six brain metastasis, five bone metastasis, one contralateral lung metastasis, one mediastinal lymph node metastasis, and six local recurrences. The tumor progression rates at years one, two, and three were 61.90%, 85.71%, and 90.48% respectively.

**Figure 1:**
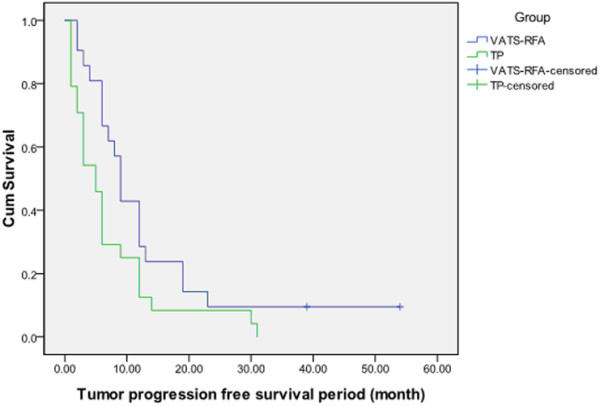
Tumor progression free survival period (month).

As for the TP group, no patient was free of tumor progression. Five cases of liver metastasis, eight cases of bone metastasis, seven cases of brain metastasis, one case of ipsilateral lung dissemination, and three cases of multi-organ metastasis occurred during the three-year follow-up. The tumor progression rate at years one, two, and three were 75%, 91.67%, and 100%, respectively. The median tumor progression-free survival time in the R-TP group was longer than that in the TP group (9.5 months versus 5.5 months, *p* = 0.028; Figure 1).

**Figure 2:**
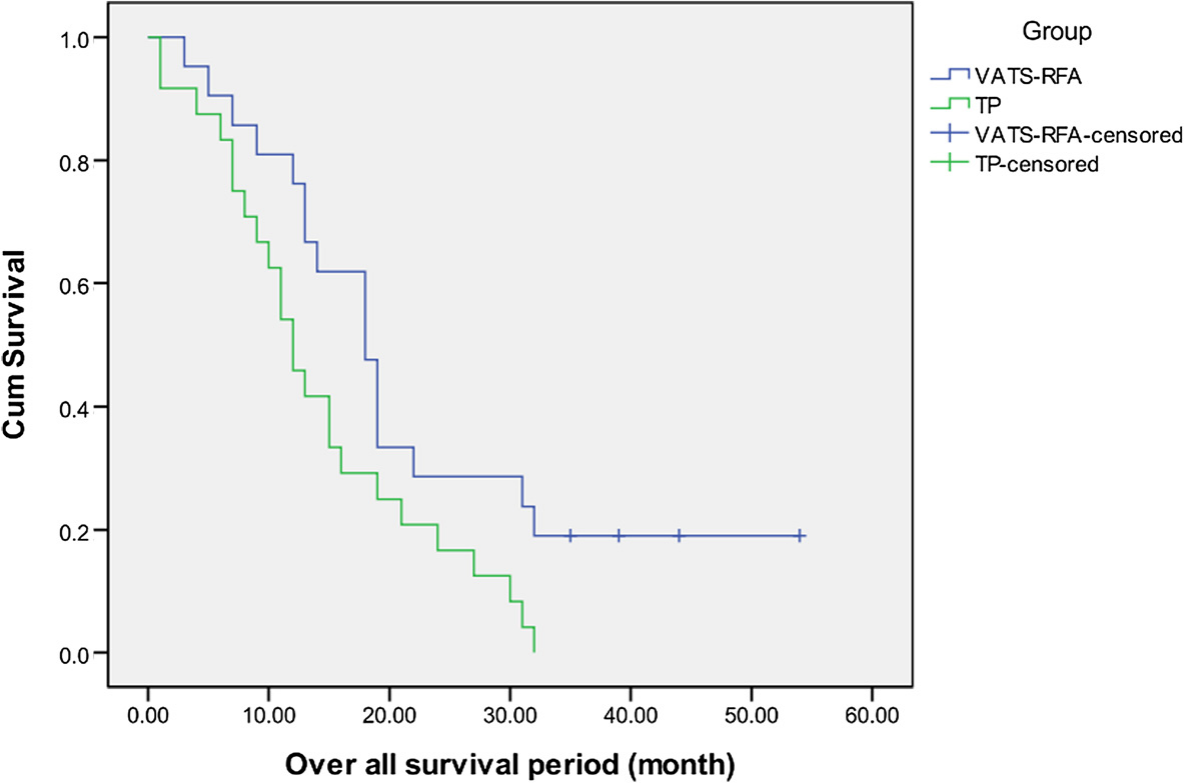
Overall survival period (month).

In the R-TP group, the overall survival rates at years one, two, and three were 80.95%, 28.57%, and 14.29%, respectively; the median survival period was 19 months. In the TP group, the overall survival rates at years one, two, and three were 54.17%, 20.83%, and 0%, respectively, and the median survival period was 12 months. Therefore, the median survival period was longer in the R-TP group than in the TP group (19 months versus 12.5 months, *p* = 0.045; Figure 2).

## Discussion

Concerning patients with early-stage NSCLC, surgical resection is the standard treatment. Some large-sample studies showed reasonable outcomes of lobectomy or pneumonectomy for selected patients with early-stage NSCLC [10]. Unfortunately, in cases of pleural dissemination, found via operation, surgery is not preferred and radiotherapy or chemotherapy is not effective either. Given that pleural dissemination has been associated with poor prognosis, it was upstaged to M1a in the seventh edition of the TNM staging classification [11]. Watanabe et al. [12] reported that more than half of all patients who underwent exploratory thoracotomy were found to have pleural dissemination. Hence, preventing pleural dissemination remains a challenge for surgeons.

Since Dupuy et al. [5] first reported RFA for the treatment of lung cancer in 2000, it has been shown to obtain positive outcomes, encouraging more studies on its application for lung cancer patients [13]. Previously, the RAPTURE study reported the application of percutaneous RFA in inoperable early-stage lung cancer; the one- and two-year survival rates of the patients were 92% and 73%, respectively [14], which were close to the values obtained by surgical resection. Indeed, RFA is an alternative treatment for patients with early-stage cancer who are inoperable. Based on these findings, we explored its further application in advanced lung cancer.

In our study, the overall survival rates at years one, two, and three were 80.95%, 28.57%, and 14.29%, respectively, and the tumor progression-free and overall survival rates were higher in the R-TP group, indicating a better prognosis than that of the patients who underwent TP only. Moreover, the application of RFA in combination with TP did not increase the risks of perioperative morbidity and mortality according to our data. Therefore, the combined R-TP treatment of pleural disseminated NSCLC does not increase the risks of higher morbidity.

According to the review by Zhu et al. [15], the complete tumor control rates by RFA ranged from 38–97%. Simon et al. [13] reported that the results were poorer in lesions that measured ≥ three centimeters. The total effective rate was 90% in the R-TP group in our research. The relatively high CR rate was realized using VATS, which allowed precise tumor localization without interference from respiration and damage caused by puncture needles. As for large tumors, multiple repositioning of the needle in different sites within the tumor was required to ensure complete ablation of the lesion.

It was concluded that NSCLC patients with malignant pleural diffusion would not benefit from tumor resection [2]. However, NSCLC patients who underwent partial resection combined with parietal pleurectomy or intrathoracic hyperthermo-chemotherapy [16-18] had good prognosis. The application of the combined treatment modality is restricted to operable patients. In contrast, video-assisted RFA can be applied more widely and preserves normal lung tissue maximally [19]. Goldberg et al. [20] used an animal model to demonstrate that normal lung tissue rapidly heals from thermal injury. Thus, for NSCLC patients with pleural dissemination, especially those with poor lung function, video-assisted RFA combined with pleurodesis is a potential alternative treatment that yields more benefits in a single surgical setting.

In our experience, when a tumor is close to large vessels, the blood may absorb a great amount of heat and impair the effectiveness of RFA. In case the lesion is close to the hilum, RFA may cause complications such as bronchopleural fistula. In these conditions, RFA is not recommended, but rather refined techniques are required to overcome these difficulties.

The common complications of CT-guided percutaneous RFA include pneumothorax and pleural effusion (range, 15.2%–55.6%), which were usually self-limiting. Approximately 3.3–38.9% of the patients required chest tube insertion for drainage [15,21]. In our study, only two complications (9.52%) occurred in the R-TP group. Although the study was based on small population, the relatively low incidence of complication suggested that thoracoscopic guidance could be considered as a safe and easy alternative to conventional therapy. The electrode tract bleeding was controlled by increasing the output power of the RITA system for electric coagulation homeostasis; in case of small branches of pulmonary artery bleeding, we used carbasus to stanch the blood. These manipulations decreased the risk of complications caused by percutaneous RFA such as pneumothorax and hemothorax. Our study showed that the perioperative characteristics did not differ between the R-TP and TP groups. One patient had pneumonia, and another complained of emptysis after surgery due to the large tumor near the central airway. The latter was eventually cured with hemostasis. Furthermore, thoracoscopic RFA did not increase the surgery-related risk and the period of postoperative hospital stay.

## Conclusions

In summary, this study evaluated 45 consecutive patients with pleural disseminated NSCLC. The perioperative and long-term follow-up results verified that the video-assisted RFA was a safe, feasible, and effective treatment for selected NSCLC patients with pleural dissemination, as well as an alternative palliative treatment for local tumor. However, further prospective studies on the role of RFA in the multimodality treatment for inoperable patients are necessary.

## Competing interests

The authors declare that they have no competing interests.

## Authors’ contributions

YXS and MZ are co-first authors, and they made equal contributions to the work. WJ designed the study. HW participated in the follow-up part of this research. HF and QW performed the operation. All authors read and approved the manuscript.

## Authors’ information

Dr Yaxing Shen^1^ and Dr Ming Zhong^2^ is the co-first author.

## Pre-publication history

The pre-publication history for this paper can be accessed here:

http://www.biomedcentral.com/1471-2482/13/19/prepub
